# Life on UNESCO Heritage: Modelling Lichen Colonization on an Armenian Cross-Stone (Khachkar)

**DOI:** 10.3390/jof12080547

**Published:** 2026-07-23

**Authors:** Pier Luigi Nimis, Sebastiano Dose, Elena Pittao, Naira Sargsyan, Lucia Muggia, Arsen Gasparyan

**Affiliations:** 1 Department of Life Sciences, University of Trieste, Via L. Giorgieri 10, 34127 Trieste, Italy; nimis@units.it (P.L.N.); sebastiano.dose@studenti.units.it (S.D.); epittao@units.it (E.P.); 2 Lichen Research and Conservation Group, Institute of Botany After A. Takhtajyan State Non-Commercial Organization, 1 Acharyan Str., Yerevan 0063, Armenia; sargsyannara259@gmail.com (N.S.); arsen.h.gasparyan@aua.am (A.G.); 3 Acopian Center for the Environment, American University of Armenia (AUA), 40 Baghramyan Str., Yerevan 0019, Armenia; 4 Zaven P. and Sonia Akian College of Science and Engineering, American University of Armenia (AUA), 40 Baghramyan Str., Yerevan 0019, Armenia

**Keywords:** bioweathering, chromatic alteration, eutrophication, lichenized fungi, Noratus cemetery, vegetation

## Abstract

This study investigates lichen colonisation on a medieval basalt Armenian cross-stone (khachkar) in the Noratus cemetery, the largest extant concentration of memorial khachkars in Armenia, integrating fine-scale vegetation sampling, microhabitat assessment, and multivariate analysis. A preliminary survey of c. 30 cross-stones confirmed that the selected one was representative of the site. Thirty-six evenly spaced relevés of 10 cm^2^ were recorded across the surface, with lichens identified macroscopically in situ and supported by laboratory-verified reference material from outside the protected area. Microhabitat descriptors were recorded, and ecological preferences were assessed using indicator values. The main environmental drivers at Noratus appear to be high solar irradiation, pronounced aridity and especially elevated nutrient deposition. Numerical classification and ordination revealed three main lichen communities, structured primarily by micro-scale water availability, exposure, and nutrient enrichment, which occupy different portions of the monument. The absence of endolithic lichens, together with the dry–cold climate of Noratus and the resistance of basalt to bioweathering, suggests limited structural impact. However, pronounced chromatic alteration was observed, producing a characteristic polychromy on the cross-stones. Lichen removal is likely to be problematic due to the extent of the cemetery, the prevalence of vegetative reproduction, and rapid recolonisation of nitrophilous species. This is the first study ever carried out on lichen colonisation of Armenian cross-stones, which are inscribed on the UNESCO List of the Intangible Cultural Heritage of Humanity. The results provide a baseline for future monitoring and support a conservation approach balancing material risk assessment with aesthetic considerations.

## 1. Introduction

Armenian khachkars are carved cross stones of considerable cultural and historical value [1,2,3]. Traditionally hewn from a single block and centred on the representation of the cross, khachkars commonly bear additional motifs such as rosettes, interlaces and botanical carvings [4]. Often set on a plinth or rectangular base and clustered near monasteries or in cemeteries, they form an open-air museum of stone-carving traditions [5,6]. Khachkars, together with their symbolism and craftsmanship, have been inscribed on the UNESCO Representative List of the Intangible Cultural Heritage of Humanity [7]. The exposed surfaces and intricate reliefs of khachkars create a mosaic of microhabitats that favour colonisation by cryptogams, particularly lichens. Lichens are important ecological pioneers on rock substrates and act as agents of both visible aesthetic change and slow biogeochemical weathering [8,9,10,11,12]. Understanding the distribution and composition of lichen communities on khachkars, therefore, addresses two complementary needs: documentation of biodiversity in a culturally significant setting and evidence-based guidance for heritage conservation and management.

Although lichen colonisation of stone monuments has been well studied in several parts of Europe [13,14,15,16,17], documentation for semi-arid regions remains limited. Existing studies from neighbouring regions include work in Iran [18,19,20], Turkey [21,22] and the Middle East [23,24]. Despite frequent and often heavy lichen colonisation of khachkars, particularly in the less arid parts of Armenia, no study has previously focused on these monuments. This study begins to fill that gap by developing a detailed, spatially explicit model of lichen distribution on a single representative khachkar from the Noratus cemetery, the largest extant collection of khachkars in Armenia. Here the specific objectives are to (i) map lichen cover on the monument and describe the principal lichen communities; (ii) relate colonisation patterns to microhabitat variables, including aspect, sheltering and surface condition; and (iii) assess whether the observed lichen communities pose a measurable risk to the structural integrity of the khachkar and discuss implications for conservation practice.

By combining fine-scale mapping with microhabitat analysis, the work aims to provide baseline data that can inform both biodiversity inventories and practical conservation strategies for khachkars and similar stone heritage in semi-arid landscapes.

## 2. Materials and Methods

### 2.1. Study Area

**Figure 1:**
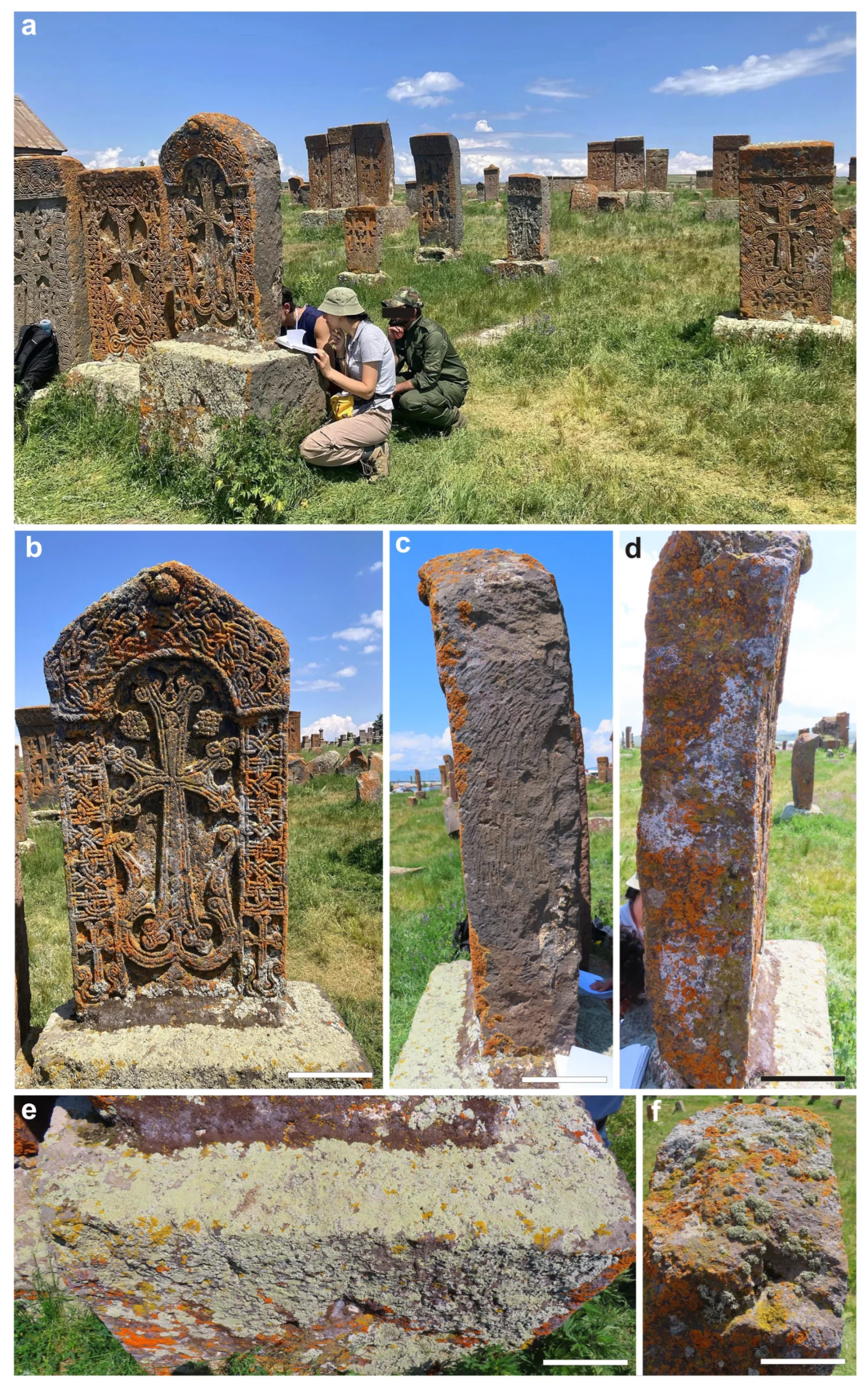
The Noratus cemetery: (**a**) a view of the cemetery area; the studied cross-stone seen from (**b**) its carved W-facing surface, (**c**) its S-facing surface, (**d**) its N-facing surface, (**e**) its horizontal surfaces of the basement, (**f**) its N-exposed surface of the upper part. Scale bars: (**b**) 20 cm; (**c**–**f**) 15 cm.

The study was carried out in the largest and most remarkable extant concentration of memorial khachkars in Armenia, located near the village of Noratus in Gegharkunik Province (Figure 1a).

The cemetery lies on the right bank of the Gavaraget River, c. 5 km northeast of Gavar, at an altitude of about 1.900 m. Noratus provides a dense, readily accessible assemblage of monuments that span a range of exposures and carving styles, making it an ideal setting in which to examine how lichen communities establish and persist on carved stone in a genuine heritage context. Some khachkars in the cemetery date to as early as the ninth century, while the majority were produced between the 13th and 17th centuries. The Noratus cemetery extends over approximately 1.63 ha and hosts nearly one thousand khachkars, each distinguished by unique compositions, carvings and ornamental details; the site also contains the small Church of St Astvatsatsin, itself of ninth-century origin [25,26]. To our knowledge, no extensive removal of lichens was previously attempted.

Gegharkunik Province has a temperate, mountainous climate strongly influenced by its elevation and by proximity to Lake Sevan. Mean annual air temperatures in lakeshore areas range from 4 to 6 °C, with mean July temperatures of 15–17 °C; extreme summer maxima may reach 33–34 °C, while January is the coldest month with mean temperatures around −5 to −6 °C. Relative humidity is lowest in September (c. 60–65%) and highest in February (c. 69–77%). Annual precipitation averages approximately 638 mm across the province, decreasing to about 487 mm in the coastal zone of Lake Sevan. The regional wind regime is dominated by westerly to southerly components, with mean wind speeds of 2–4 m s^−1^ [27]. These climatic and topographic gradients generate marked microclimatic variation across the cemetery, which in turn contributes to the diversity of microhabitats available for cryptogams.

The cross stone selected for detailed sampling (Figure 1b) is representative of the medieval khachkars present at Noratus (see later). Located at 40°22′26.79″ N, 45°10′51.33″ E, it is carved from basalt and dates to the 13th–14th centuries. The monument comprises a pedestal measuring 88 × 57 cm and a 21 cm thick, 61 cm wide and 121 cm high stele with a domed upper cornice. The largest faces of the stele are oriented east–west; the west-facing side is carved while the east-facing side is smooth. Its material, relief complexity and exposure make it a suitable exemplar for spatially explicit mapping of lichen cover and for relating colonisation patterns to microhabitat variables within the Noratus assemblage.

### 2.2. Preliminary Surveys

Prior to detailed sampling, a visual survey of lichen cover was carried out on c. 30 cross stones within the Noratus cemetery to test for possible heterogeneity of lichen colonisation (Figure 1). Species assemblages and vegetation patterns proved to be homogeneous on medieval cross stones across the area. On this basis, a single khachkar was selected at random for intensive, spatially explicit sampling (Figure 1b–f). A further preliminary survey, carried out on the carved flanks of 3 cross-stones, aimed at verifying whether consistent differences in lichen colonisation were present between the carved and projecting parts of the monument. The results indicated no relevant difference.

### 2.3. Vegetation Sampling and Species Identification

**Figure 2:**
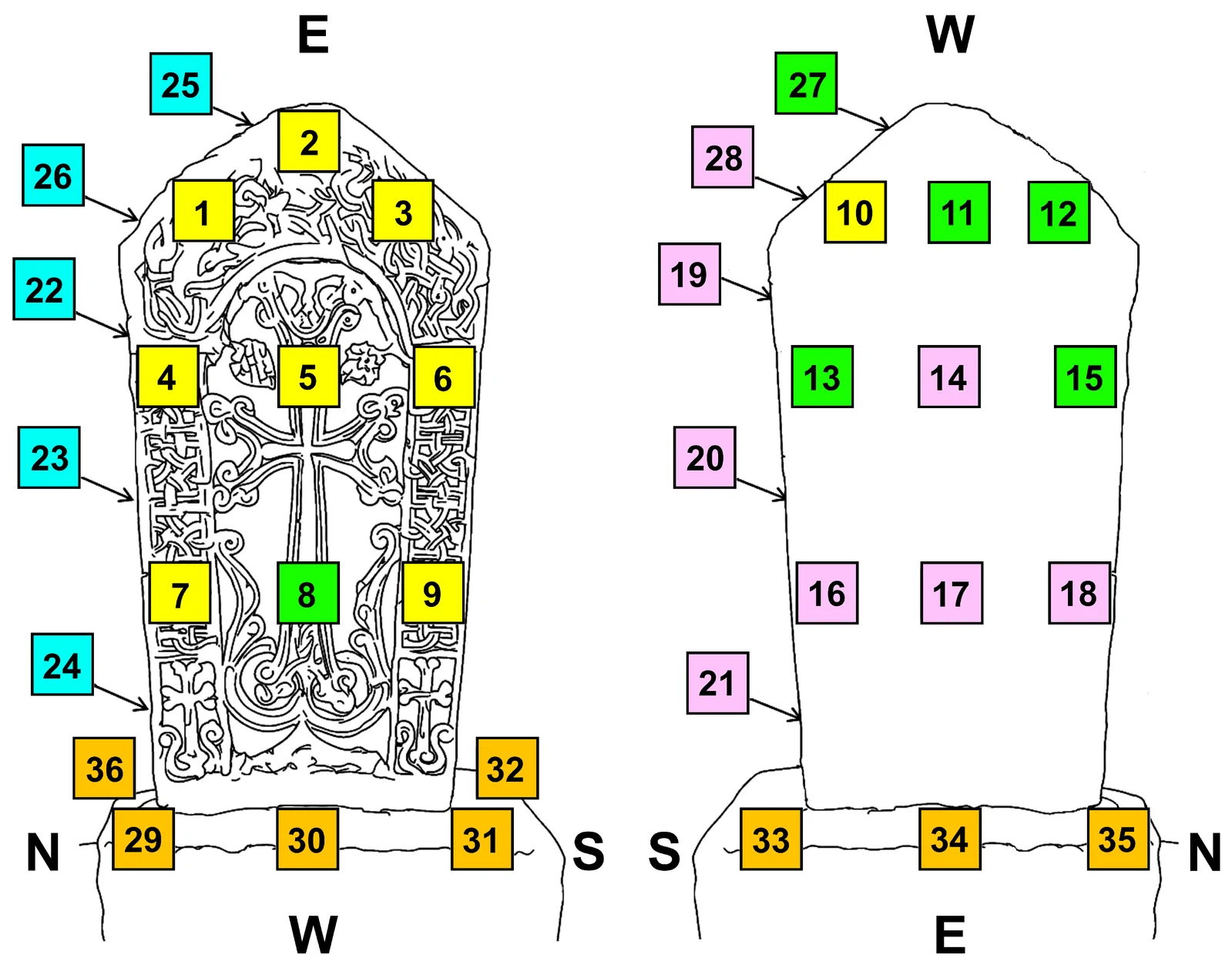
Location of the relevés on the cross-stone. The colours refer to the five relevé groups obtained by classification, as in Figure 3 and Table S1.

A total of 36 relevés were sampled, equally spaced across the monument (the vertical surfaces of the pedestal were not sampled because they were partly masked by grass, which biases the development of lichen colonisation). The position of relevés on the monument is shown in Figure 2.

Each relevé listed all lichen species occurring within a standard 10 cm^2^ surface; species cover was recorded using the six-class scale of Braun–Blanquet [28], as modified by Nimis [29], for lichen vegetation: + = up to 1%; 1 = 1–20%; 2 = 21–40%; 3 = 41–60%; 4 = 61–80%; 5 = 81–100%.

For each relevé, we recorded a set of microhabitat descriptors to permit analysis of environmental controls on colonisation:

- Aspect (cardinal orientation) and inclination (vertical/horizontal);
- Degree of sheltering (exposed, partially sheltered, sheltered);
- Evidence of bird activity (presence/absence of perching or droppings);
- Macroscopic signs of biodeterioration (cracking, flaking, granular disintegration).

Because sampling took place within a protected site, no destructive sampling was permitted. Consequently, lichens were identified macroscopically in the field. To increase confidence in field identifications, the study made use of comparative material, i.e., lichens collected from natural rock outcrops outside the protected area, which were analysed in the laboratory using standard techniques and served as reference material for field identifications. Lichens were identified on the basis of the main floras concerning Europe [30,31,32,33,34,35], alongside many monographic treatments that also take into consideration extra-European taxa. Due to the scarcity of the available material, TLC analyses were not carried out for studying lichen secondary metabolites, using instead the classical spot reactions. All of these reference collections are now stored in the TSB Herbarium. Lichen nomenclature follows Nimis [36].

### 2.4. Ecological Characterisation of the Lichen Biota

The ecological characterisation of the lichen biota of the monument was based on the ecological indicator values proposed by Nimis [37], which express the ecological range of each species to aridity and eutrophication on a 5-class ordinal scale. The percent occupancies of the species present on the sampled cross–stone in each class were compared with those of a comparable, entire lichen biota of base-rich siliceous rocks in the montane belt of Italy (558 infrageneric taxa).

### 2.5. Multivariate Analysis

The species × relevé matrix was subjected to numerical classification for detecting groups of relevés with similar lichen vegetation and to ordination for revealing underlying ecological gradients. The R 4.3.0 software [38] was used after loading the ‘amap’ package version 0.8.19 [39]. Classification was carried out on maximum values of percentage cover for each class, using Euclidean Distance and the linkage method ‘ward.D2’ (i.e., Ward’s minimum variance method) for relevés, while for species Correlation Coefficient, i.e., ‘Centered Pearson’ 1 − corr(x,y) and Complete Linkage clustering were used. To test whether the relevé groups obtained by classification were mainly due to a few species with high cover, presence–absence data were used for the ordination (Principal Component Analysis), which was performed using the function ‘prcomp’.

## 3. Results

### 3.1. Preliminary Survey and Representativeness of the Selected Monument

The preliminary survey revealed that, of the 30 examined cross-stones, only one specimen supported a clearly different lichen assemblage, likely due to a different lithology (more acidic siliceous rock), including *Dimelaena oreina*, *Lecanora argopholis*, *L. cenisia* and *Rhizoplaca chrysoleuca*. All other examined khachkars showed broadly comparable species composition. Therefore, the selected cross-stone is considered representative of the prevailing lichen colonisation pattern across the Noratus cemetery.

### 3.2. Species Composition and Ecological Structure

A total of 27 lichen species were recorded on the sampled monument, of which 23 were identified at the species level (Table S1). Growth forms were dominated by crustose lichens (78%), while four species were foliose and two were fruticose. All taxa were epilithic; no endolithic species were observed. Regarding reproductive strategies, 63% of species reproduce sexually, whereas 37% propagate asexually via soredia.

**Figure 3:**
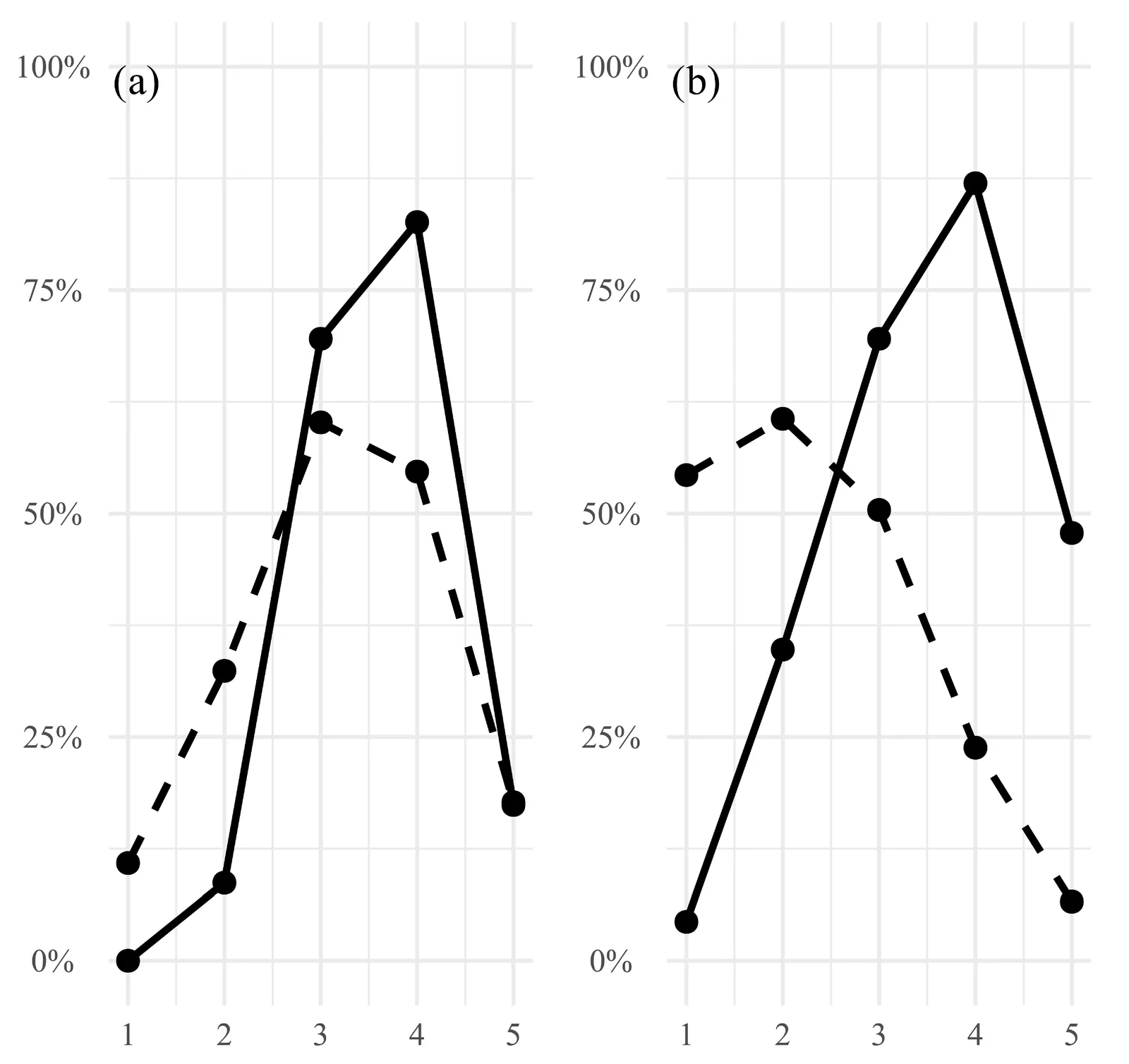
Percent occupancies of the species in the five classes of the indexes of aridity (**a**) and eutrophication (**b**). Continuous line: lichens of the Noratus cross-stone; dotted line: entire biota of lichens from base-rich rocks in the montane belt of Italy. The indexes range from 1 (very humid/no eutrophication) to 5 (extremely high aridity/eutrophication).

Ecologically, the lichen biota are shifted towards moderately high aridity conditions and especially towards high eutrophication levels (Figure 3).

### 3.3. Community Classification of Relevés

**Figure 4:**
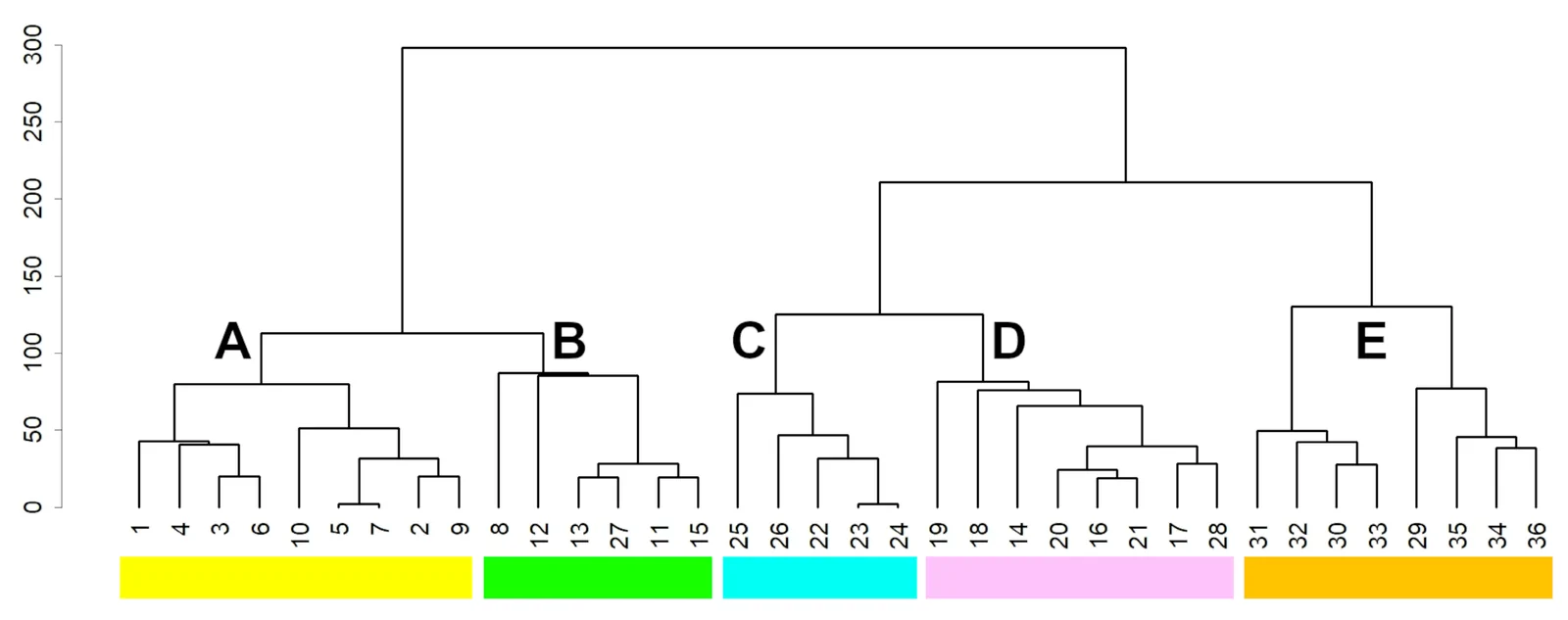
Dendrogram of relevés, numbered as in Table S1. The five main relevé groups are designed by letters and colours, as in Figure 2 and Table S1.

Cluster analysis of relevés (Figure 4) identified five main groups (A–E), whose spatial distribution on the monument is shown in Figure 2.

These groups are characterised as follows:

- Group A (nine relevés; mean cover 75%): Dominated by *Rusavskia elegans*, *Candelariella vitellina* and *Acarospora umbilicata*. It occurs mainly on west-facing vertical surfaces. The dominant colour is orange (Figure 1b).
- Group B (six relevés; mean cover 71%): Similar to Group A but with reduced cover of *Acarospora umbilicata* and *Candelariella vitellina*. It is mainly found on the upper parts of east-facing vertical surfaces. The dominant colour remains orange.
- Group C (five relevés; mean cover 87%): Characterised by the presence of *Ramalina polymorpha* and *R. capitata* (the only fruticose taxa), together with nitrophilous foliose species such as *Physcia caesia* and *Phaeophyscia orbicularis*. It occurs on north-facing flanks and the uppermost part of the monument. Dominant colours are greenish and whitish (Figure 1d,f).
- Group D (eight relevés; mean cover 32%): No diagnostic species were identified; this group is characterised by low overall cover. It occurs on south-facing flanks and lower parts of east-facing surfaces. The rock colour remains visually dominant (Figure 1c).
- Group E (eight relevés; mean cover 87%): Dominated by *Protoparmeliopsis muralis*, with low abundance of *Rusavskia elegans* and presence of *Lecidella carpathica*, *Acarospora fuscata* and *Protoparmeliopsis garovaglii*. It is restricted to horizontal surfaces of the pedestal. The dominant colour is pale yellowish-green (Figure 1e).

### 3.4. Ordination of Relevés

**Figure 5:**
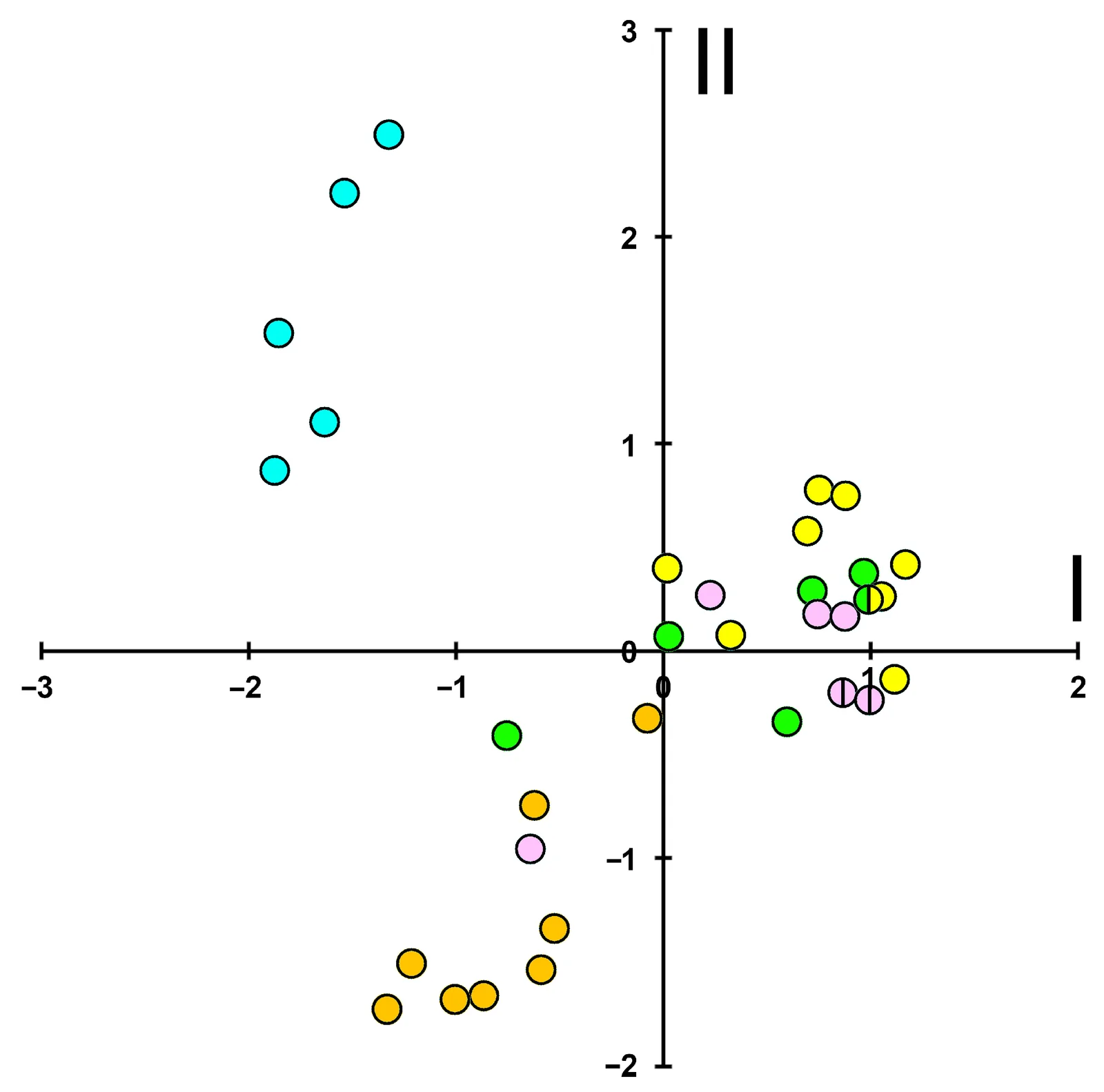
Ordination of relevés. The five main relevé groups are designed by colours, as in Figure 2 and Figure 4, and Table S1. Markers split in half indicate two relevés with identical scores.

Ordination analysis (Figure 5) showed that the first axis (25% of variance) separates Groups C and E (negative scores) from Groups A, B and D (positive scores).

The second axis (18% of variance) further distinguishes Group C (positive scores) from Group E (negative scores). Compared with the classification results, Group D shows a different position in ordination space, clustering closer to Groups A and B when presence–absence data are used.

### 3.5. Synthesis of Community Types

Combining classification and ordination results, three main lichen community types are recognised:

1. *Ramalina polymorpha*-community (Group C), restricted to north-facing surfaces;
2. *Protoparmeliopsis muralis*-community (Group E), restricted to horizontal pedestal surfaces;
3. *Rusavskia elegans*-community, comprising three variants (Groups A, B and D) and occupying all sun-exposed vertical surfaces.

## 4. Discussion

### 4.1. Patterns of Lichen Growth and Their Ecological Drivers

All of the identified lichen species have a very broad, sometimes holarctic or subcosmopolitan distribution, also occurring in Europe. This broad biogeographical affinity indicates that colonisation is driven primarily by regional species availability and by local environmental filters rather than by a unique, locally endemic flora. The dominant environmental drivers at Noratus appear to be high solar irradiation, pronounced aridity and especially elevated nutrient deposition, the latter most plausibly arising from aeolian transport of dust and agricultural fertilisers from surrounding cultivated land and from bird activity.

While high eutrophication affects the entire lichen biota of the monument, micro-scale water availability emerges as the principal factor structuring lichen distribution. North-facing surfaces retain moisture for longer periods and therefore support humidity-demanding communities, whereas sun-exposed, rain-protected vertical faces remain comparatively dry and are dominated by stress-tolerant taxa. Bird activity also modifies local nutrient inputs and thus influences community composition, particularly on upper surfaces used as perches. Three main community types can be distinguished on the monument:

- North-exposed flanks (group C). These are colonised by a community corresponding to *Ramalinetum capitatae* [40], a nitrophilous to ornitocoprophilous assemblage typical of rain-exposed siliceous rocks and widely reported across Europe [41,42,43,44,45]. On khachkars, bird perching increases local nutrient input and, together with reduced insolation, creates conditions favourable to fruticose and foliose lichens that require higher humidity, these taxa being therefore absent from the much drier south-facing flanks.
- Horizontal pedestal surfaces (group E). These are dominated by *Protoparmeliopsis muralis* subsp. *muralis*, a widespread, subcosmopolitan, nitrophilous lichen that is common on nutrient-enriched rocks, including anthropogenic substrates, also in urban areas across Europe and beyond [46]. On the monument it mainly occupies horizontal surfaces that receive direct rainfall and episodic nutrient flushing.
- Sun-exposed vertical surfaces (groups A, B and D). These are dominated by the *Rusavskia elegans* community, a vegetation type reported from northern and central Europe, the Alps and the Mediterranean mountains [42,47,48,49]. It establishes on base-rich rocks in strongly nutrient-enriched, sunny and dry microsites, often on vertical faces or under overhangs where rain impact is limited. Three variants are evident on the khachkar: (1) a relatively species-rich variant with the highest cover (group A), mainly on the west-facing side and likely benefiting from occasional humidity delivered by westerly winds; (2) a species-poorer variant with lower cover (group B), occupying the drier upper part of the east-facing surface; and (3) a very species-poor variant with the lowest cover (group D), restricted to the driest surfaces (south-facing flanks and the lowermost east-facing vertical surfaces).

The three dominant species, while being all strongly nitrophilous, have slightly different ecological requirements [37]: *Ramalina polymorpha* is bound to more humid conditions, and *Rusavskia elegans* to drier conditions, being also able to thrive on rain-protected surfaces under overhangs, while *Protoparmeliopsis muralis* is less xerophilous and thrives on surfaces wetted by rain. These patterns underline the importance of fine-scale topography and orientation in creating a mosaic of microhabitats on a single monument, and they emphasise that conservation assessments must be considered within-monument heterogeneity rather than treating a khachkar as a uniform substrate.

### 4.2. Bioweathering of Basalt

As far as the lichen-induced biodegradation of the basaltic surfaces of the cross-stones are concerned, a positive fact is the total absence of endolithic lichens on the monument, which means that the fungal hyphae of all species do not penetrate into the rock. However, the potential, also for epilithic lichens, to drive biogeochemical weathering of basalt is well documented experimentally. Primary basalt minerals such as pyroxene, olivine and feldspar are susceptible to attack by organic acids released by lichens [50], with mobilisation of Fe and Mg often exceeding that of Ca and Al [51]. Such processes can lead to secondary coatings (e.g., of hydrous ferric oxides) and to the formation of amorphous alteration products [52,53]. Field and laboratory studies also show that porous, glassy or chemically altered basalts may exhibit significant biofilm and lichen-driven alteration [54,55].

However, the rate and intensity of bioweathering are strongly climate-dependent [56,57]. In warm, humid environments these processes proceed more rapidly [58], while in the dry–cold climate of Noratus they are likely to be much slower. Moreover, lichens can even exert protective effects on stone surfaces, as they may reduce direct wind and rain impact [59,60]; retain moisture within the superficial biofilm and thallus (thereby moderating thermal fluctuations, see [61]; and limit salt and pollutant ingress [62,63]. Pinna has emphasised that, in some contexts, lichens can reduce erosion by lowering the amount of water penetrating the rock, also decreasing the damage by atmospheric agents such as wind, rain, pollutants, and salt aerosol [64].

On the Noratus khachkars the carved motifs remain sharply recognisable despite centuries of lichen cover, suggesting that, at the present climatic regime and on a relatively resistant lithology such as basalt, lichen growth has not produced obvious structural loss of the carvings. This observation supports a context-dependent balance: lichens have the potential to contribute to mineral mobilisation and surface alteration, but under the prevailing semi-arid, cold conditions and on basalt, the net structural impact appears limited. Nevertheless, slow chemical alteration may be occurring at scales not yet visible macroscopically; long-term monitoring and targeted microchemical analyses would be required to quantify such processes.

### 4.3. Chromatic Alterations

While structural integrity appears largely preserved, lichens are responsible for pronounced chromatic alteration across the Noratus cemetery. Comparable studies have documented lichen-driven colour changes on monuments [14,65], with variable conservation implications depending on the site and the values at stake. At Noratus, the typical chromatic pattern is consistent: vertical surfaces appear bright orange owing to dominance by *Rusavskia elegans*, pedestals contrast with a pale greenish-yellow hue dominated by *Protoparmeliopsis muralis*, and north-facing surfaces show paler, whitish or greenish tones. The originally monochromatic cross stones therefore now present a strongly polychromatic appearance. Chromatic change is primarily an aesthetic and interpretative issue rather than an unequivocal material emergency. In some heritage contexts, lichen-induced colouration is judged to obscure important visual contrasts or original finishes [8], while in others it is accepted as part of the site’s character and history [66]. At Noratus, the brightly coloured khachkars have become a distinctive feature of the cemetery; any management decision must therefore weigh the aesthetic and cultural values associated with lichen cover against potential long-term material risks.

### 4.4. Problems in Lichen Removal

There are several factors that could complicate the removal of lichens from the cross-stones of Noratus. Firstly, many of the species present reproduce by vegetative propagules (soredia) that are readily detached and dispersed by mechanical disturbance and wind [67]. Mechanical scraping, brushing or blasting of lichen thalli tends to leave numerous propagules on the stone surface or in adjacent microhabitats, which are subsequently redistributed by wind, rain splash or human activity, thereby promoting rapid re-colonisation and rendering removal potentially counterproductive [68]. Secondly, the widespread application of biocides [69] in an area frequented by tourists is not advisable, while ostensibly more environmentally benign methods, such as heat-shock treatments [70], are likely to be less effective under the relatively low ambient temperatures at the site. A further complication arises from the fact that almost all lichens occurring on the cross-stones are associated with pronounced eutrophication. Empirical evidence indicates that recolonisation of stonework by lichens can occur within months to a few years after cleaning when environmental drivers—most notably nitrogen deposition and local nutrient sources—remain unaltered [14,66,71], which undermines the long-term efficacy of restorations. Moreover, although lichen removal from monuments is widely practised, it can damage the substrate [72,73] and, in the case of extensive or repeated biocide use, the surrounding environment.

Accordingly, for the Noratus cemetery, we concur with Sheppard [74], who, rather than advocating mechanical or biocidal cleaning—both of which entail substantial drawbacks—recommends a policy of minimal intervention, favouring non-destructive documentation and recording of the monuments and allowing lichens to contribute to the site’s aesthetic.

## 5. Conclusions

Lichen colonisation of the Noratus khachkars is governed by microhabitat water availability, exposure and especially nutrient input, producing a predictable spatial pattern of communities. Although lichens can mediate mineral mobilisation in basalt, the current evidence suggests limited structural impact under the site’s dry–cold climate. The most conspicuous effect is chromatic alteration, which, however, has become an intrinsic characteristic of the cemetery. Conservation responses should therefore be proportionate, prioritising monitoring and targeted action only where material risk is demonstrated, while recognising the cultural and aesthetic dimensions of lichen cover.

## Data Availability

The original contributions presented in this study are included in the article/Supplementary Material. Further inquiries can be directed to the corresponding authors.

## Supplementary Materials

The following supporting information can be downloaded at https://www.mdpi.com/article/10.3390/jof12080547/s1. Table S1: Matrix of relevés and species. The relevés are ordered according to their dendrogram (Figure 4), the five main relevé groups being highlighted by colours.

## Abbreviations

The following abbreviations are used in this manuscript:

UNESCO	United Nations Educational, Scientific and Cultural Organization
TSB	Herbarium of the University of Trieste
